# rNMR: open source software for identifying and quantifying metabolites in NMR spectra

**DOI:** 10.1002/mrc.2526

**Published:** 2009-12

**Authors:** Ian A Lewis, Seth C Schommer, John L Markley

**Affiliations:** National Magnetic Resonance Facility at Madison, Department of Biochemistry, University of WisconsinMadison, 433 Babcock Drive, Madison, WI 53706-1544, USA

**Keywords:** data mining, data organization, data visualization, metabolite identification, metabolite quantification, NMR-based metabolomics, region of interest, software, two-dimensional proton-carbon NMR

## Abstract

Despite the extensive use of nuclear magnetic resonance (NMR) for metabolomics, no publicly available tools have been designed for identifying and quantifying metabolites across multiple spectra. We introduce here a new open source software tool, rNMR, which provides a simple graphics-based method for visualizing, identifying, and quantifying metabolites across multiple one- or two-dimensional NMR spectra. rNMR differs from existing software tools for NMR spectroscopy in that analyses are based on regions of interest (ROIs) rather than peak lists. ROIs contain all of the underlying NMR data within user-defined chemical shift ranges. ROIs can be inspected visually, and they support robust quantification of NMR signals. ROI-based analyses support simultaneous views of metabolite signals from up to hundreds of spectra, and ROI boundaries can be adjusted dynamically to ensure that signals corresponding to assigned atoms are analyzed consistently throughout the dataset. We describe how rNMR greatly reduces the time required for robust bioanalytical analysis of complex NMR data. An rNMR analysis yields a compact and transparent way of archiving the results from a metabolomics study so that it can be examined and evaluated by others. The rNMR website at http://rnmr.nmrfam.wisc.edu offers downloadable versions of rNMR for Windows, Macintosh, and Linux platforms along with extensive help documentation, instructional videos, and sample data. Copyright © 2009 John Wiley & Sons, Ltd.

## Introduction

Nuclear magnetic resonance (NMR) spectra of biological extracts contain thousands of overlapping signals from many molecules. Translating this complex spectral data into concentrations of individual metabolites is one of the most significant challenges facing modern metabolomics. Recently, methods have been developed for identifying[1–4] and accurately quantifying[5] metabolites using multidimensional NMR and databases of metabolite standards. Although these methods are effective in small-scale studies, the significant time required for identifying metabolites, assigning individual NMR signals, and quantifying resonances has made larger scale applications of these tools impractical.

**Figure 1:**
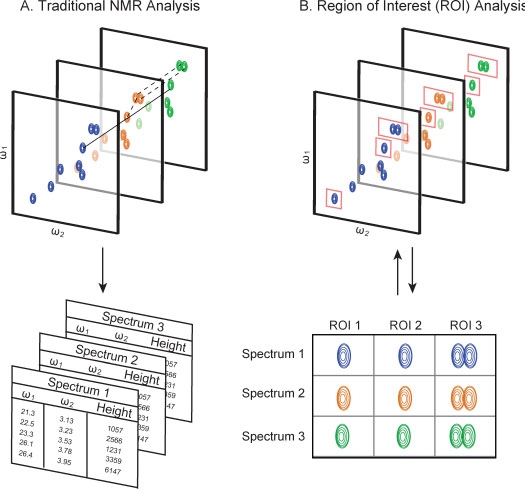
Traditional (A) *versus* ROI-based (B) analysis of two-dimensional NMR data. Traditional approaches formatching signals across multiple spectra are prone to error because of chemical shift variation. The ROI-based approach used by rNMR avoids this problem by allowing users to view all of the NMR data related to a resonance assignment, and to dynamically resize or move ROIs so that only the target signals are included in any analysis.

The main difficulties in translating quantitative metabolomics to larger scale studies result from the time-consuming nature of traditional NMR resonance assignment. Metabolomics studies often involve 50 or more metabolites, hundreds of samples, and thousands of individual resonance assignments. Moreover, the positions (chemical shifts) of NMR signals in complex biological extracts are subject to unpredictable variation caused by small differences in solution chemistry between samples.[6] The complexity of the NMR spectra and inherent variations in chemical shifts make automated peak assignment error prone when assignments are transferred from one spectrum to another (Fig. 1A). If peak matching algorithms are allowed to be flexible to chemical shift variation, then NMR peaks can be improperly matched to neighboring signals; if tolerances are too rigid, then signals can be missed completely. As a result, reliable metabolite assignments ultimately require visual inspection of the raw NMR data. Currently, none of the publicly available software tools allow NMR signals from individual metabolites to be easily compared across multiple spectra.

To make quantitative NMR-based metabolomics more feasible in large-scale studies,we have developed a simple, graphics-based method for comparing resonance assignments across multiple spectra. Our solution is based on the concept of a region of interest (ROI) (Fig. 1B). ROIs are dynamic user-defined subsections of a spectrum that can be actively moved or resized to enclose an NMR signal. In contrast to peak lists, which are static summaries containing limited information, ROIs contain all of the underlying NMR datawithin the ROI boundaries and can be rapidly inspected. We have implemented this approach in the software tool (rNMR) described here.

## Results and Discussion

### Program design objectives

rNMR was designed with four major objectives: (i) to simplify analyses of multiple NMR spectra, (ii) to provide a transparent mechanism for connecting quantitative summaries with the underlying NMR data, (iii) to provide a customizable framework for developing new NMR software tools, and (iv) to create a userfriendly program for analyzing NMR spectra. We developed rNMR as an add-on package for the R statistical software environment (freely available from http://www.r-project.org) because R is inherently suited to these objectives. Programs written in R provide direct access to the code and data tables. Users can insert custom functions, viewandmodifythedata,andredirectexisting functions for other purposes. These manipulations can be performed at any time within the main R console. Furthermore, R is supported by extensive public libraries formathematics, statistics, and graphics. These tools can be easily integrated into existing rNMR functions or can be applied ad hoc as the need arises. Any modifications can be readily shared with the community because rNMR's licensing (GPLv3; http://www.gnu.org) gives users the freedom to modify and redistribute the program.

### Batch manipulations

**Figure 2:**
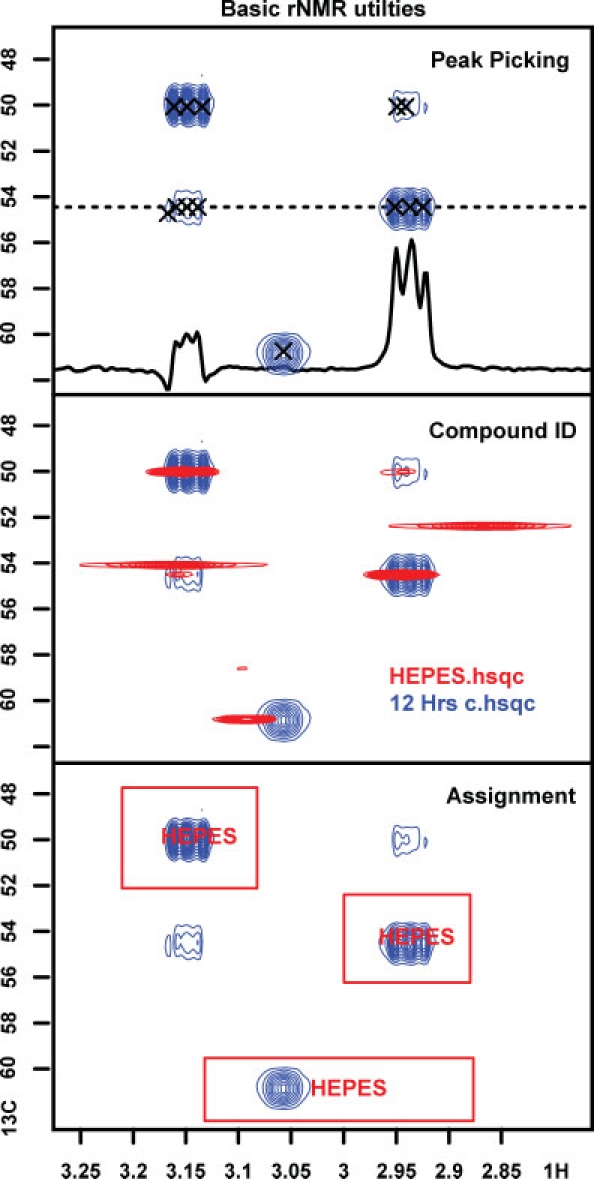
The rNMR main plotting window illustrating several basic features of the program: peak picking, one-dimensional slice visualization, spectral overlay, and ROI-based assignment.Metabolites can be identified by submitting peak lists generated by rNMR to the MadisonMetabolomics Consortium Database (http://mmcd.nmrfam.wisc.edu). These automated identifications can be verified by overlaying spectral standards available from the BioMagResBank (http://www.bmrb.wisc.edu). Resonance assignments are thus based on ROIs enclosing each target resonance. Once ROIs have been generated, data from multiple spectra can be visualized simultaneously (Fig. 3) and quantified.

To simplify analyses of multiple one- or two-dimensional NMR spectra, all of rNMR's basic tools were designed to support batch operations. Basic rNMR tools include functions for overlaying spectra, displaying slices and projections of two-dimensional spectra, adjusting chemical shift referencing, peak picking, graphics settings, and a variety of plotting methods (Fig. 2). These functions can be applied in batch to any of the open spectra via point-and-click graphical user interfaces or line commands. Moreover, settings designated for one spectrum can be transferred directly to other spectra because rNMR's functions are designed to operate independently of the NMR acquisition parameters. Peak picking thresholds and contour levels, for example, are defined by standard deviations above the thermal noise.

### ROI-based tools

**Figure 3:**
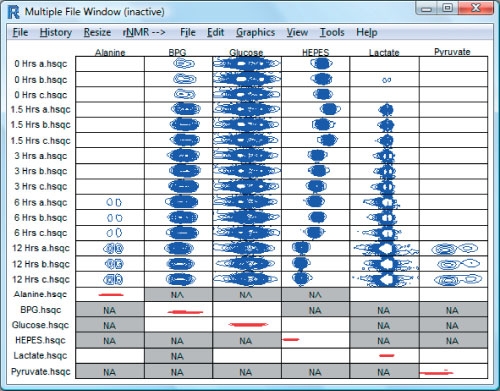
A screenshot from the rNMR multiple-file window showing ROIs containing two-dimensional NMR signals (blue) from 6 molecules observed in 15 spectra of red blood cell extracts incubatedwith [U-^13^C]-glucose. Spectra correspond to aliquotswithdrawn over a 12-h time course. The red peaks are from small molecule standards corresponding to each of the observed metabolites. 4-(2-hydroxyethyl)-1-piperazineethanesulfonic acid (HEPES), which was included in the incubation medium, serves as a monitor of pH; its chemical shift can be used to determine the pH of each aliquot. If an ROI falls outside the chemical shift range of a spectrum, rNMR displays a gray box with the designation ‘NA.’.

The ROI-based tools included inrNMRprovide a simple mechanism for visualizing NMR data across multiple spectra. ROIs can be imported from a tab delimited file, designated automatically or generated manually by clicking on a desired region of a spectrum. Once ROIs have been created (Fig. 2), data can be extracted from hundreds of files and displayed side by side in a multiple-file graphics window (Fig. 3). This strategy allows gigabytes of raw NMR data to be visually inspected in a few minutes and allows assignment errors detected at any stage of an analysis to be corrected by simply adjusting the ROI boundaries. Because rNMR requires assigned resonances to fall within a defined chemical shift range, and because each spectrum is subjected to the same assignment criteria, rNMR enforces consistent assignments across samples while maintaining flexibility for variation in chemical shift (Fig. 3).

In addition to simplifying resonance assignments, ROI-based analyses provide a transparent link between quantitative analyses and the underlying NMR data. Quantification is based directly on the NMR data displayed in the ROI windows. The NMR signal behind any data point can be visually inspected by simply clicking on the appropriate ROI. Because all rNMRanalyses are regenerated on the fly from raw NMR data and a table containing the chemical shift boundaries of each ROI, any rNMR analysis can be duplicated and rigorously evaluated by other researchers. To illustrate this point, we have provided (at http://rnmr.nmrfam.wisc.edu) the NMR data, ROI table, and instructions detailing the three-step process required to reproduce Fig. 3.

### rNMR user interface

One of our major objectives in designing rNMR was to provide a user-friendly interface for new users while providing more advanced users an easy mechanism for modifying the program. For new users,we have created a suite of point-and-click graphical tools that are accessible from drop-down menus. Although these tools provide direct accesses to all of rNMR's features, the main objective in their design was to provide a simple interface for interacting with data. Wherever possible, we have combined or eliminated superfluous features to simplify the user interface and tominimize the number of steps required to conduct quantitative analyses.Much of this economy has beenmadepossible by rNMR's ‘what you see is what you get’ philosophy. Quantitative analyses and peak picking thresholds, for example, are based directly on the data displayed to the user. This approach has the advantages of both eliminating redundancy andmaking quantitative procedures more transparent.

To facilitate customization of rNMR, the program was designed with a pseudo object-oriented framework in which all of the plotting and quantitative functions act on a series of usermodifiable lists and tables. All of rNMR's graphics are regenerated on the fly from these objects. This strategy allows custom functions to be seamlessly integrated into the existing software. In addition, all of rNMR's features are accessible via line command and can be strung together to create standardized processing scripts.Wehave also provided a suite of utility functions that simplify customization of the program. These tools include functions for manipulating graphics settings, creating custom dialog boxes, and managing windows.

### Support for rNMR

We designed rNMR initially to solve practical problems encountered in our own research. In expanding this software for more general metabolomics applications, we have solicited feedback from rNMR beta testers around the world. The current release of rNMR has undergone more than 6 months of rigorous beta testing, and several of the features currently implemented in rNMR were suggested by outside users. We have structured rNMR to be a community maintained program, and we encourage others to modify it to meet their needs. We plan to continue supporting our version of the software and are actively expanding rNMR's capabilities with input from the user community.

## Conclusions

rNMR is an open source software tool that solves three critical problems facing modern bioanalytical metabolomics: the difficulty in analyzing one- and two-dimensional NMR data from studies involving large numbers of spectra, the combinatorial problems associated with automated resonance assignments, and the difficulty in evaluating and documenting NMR-based analyses. The rNMR software program, in combination with existing tools for identifying[1–4] and quantifying[2] metabolites in complex biological extracts, offers a robust platform for extending quantitative bioanalytical metabolomics to large-scale studies. Versions of rNMR forWindows, Macintosh, and Linux, and extensive help documentation, instructional videos, and sample data (including all of the data used to create Figs 2 and 3) are freely available from http://rnmr.nmrfam.wisc.edu.
